# Corrected QTc interval combined with troponin value and mortality in acute ischemic stroke

**DOI:** 10.3389/fcvm.2023.1253871

**Published:** 2023-09-26

**Authors:** Sung-Ho Ahn, Ji-Sung Lee, Mi-sook Yun, Jung-Hee Han, Soo-Young Kim, Sang-Hyun Lee, Min-Gyu Park, Kyung-Pil Park, Dong-Wha Kang, Jong S. Kim, Sun U. Kwon

**Affiliations:** ^1^ Department of Neurology, Research Institute for Convergence of Biomedical Science and Technology, Pusan National University Yangsan Hospital, Pusan National University School of Medicine, Busan, Republic of Korea; ^2^ Clinical Research Center, Asan Medical Center, College of Medicine, University of Ulsan, Seoul, Republic of Korea; ^3^ Division of Biostatistics, Research Institute for Convergence of Biomedical Science and Technology, Pusan National University Yangsan Hospital, Pusan National University School of Medicine, Busan, Republic of Korea; ^4^ Department of Neurology, Asan Medical Center, College of Medicine, University of Ulsan, Seoul, Republic of Korea; ^5^ Division of Cardiology, Research Institute for Convergence of Biomedical Science and Technology, Pusan National University Yangsan Hospital, Pusan National University School of Medicine, Busan, Republic of Korea; ^6^ Department of Neurology, Gangneung Asan Hospital, University of Ulsan, Gangneung, Republic of Korea

**Keywords:** ischemic stroke, electrocardiography, troponin, QTc interval, mortality

## Abstract

**Background and Purpose:**

Cardiac biomarkers including, elevated troponin (ET) and prolonged heart rate-corrected QT (PQTc) interval on electrocardiography are known to frequent and have a prognostic significance in patients with acute ischemic stroke (AIS). However, it is still challenging to practically apply the results for appropriate risk stratification. This study evaluate whether combining ET and PQTc interval can better assess the long-term prognosis in AIS patients.

**Methods:**

In this prospectively registered observational study between May 2007 and December 2011, ET was defined as serum troponin-*I* ≥ 0.04 ng/ml and PQTc interval was defined as the highest tertile of sex-specific QTc interval (men ≥ 469 ms or women ≥ 487 ms).

**Results:**

Among the 1,668 patients [1018 (61.0%) men; mean age 66.0 ± 12.4 years], patients were stratified into four groups according to the combination of ET and PQTc intervals. During a median follow-up of 33 months, ET (hazard ratio [HR]: 4.38, 95% confidence interval [CI]: 2.94–6.53) or PQTc interval (HR: 1.53, 95% CI: 1.16–2.01) alone or both (HR: 1.77, 95% CI: 1.16–2.71) was associated with increased all-cause mortality. Furthermore, ET, PQTc interval alone or both was associated with vascular death, whereas only ET alone was associated with non-vascular death. Comorbidity burden, especially atrial fibrillation and congestive heart failure, and stroke severity gradually increased both with troponin value and QTc-interval.

**Conclusions:**

In patients with AIS, combining ET and PQTc interval on ECG enhances risk stratification for long-term mortality while facilitating the discerning ability for the burden of comorbidities and stroke severity.

## Introduction

According to the Global Burden of Disease, stroke remains the predominant cause of death and disability worldwide (1), and cardiovascular complications are the second leading cause of death, followed by stroke (2). Therefore, assessment of biomarkers for cardiac injury and dysfunction, in particular serum cardiac troponin assay and electrocardiography (ECG), has been recommended for early recognition of cardiovascular abnormalities in patients with acute ischemic stroke (AIS) (3). Consequently, according to the guideline-based routine cardiac monitoring, the elevated troponin (ET, 18%–34%) (4) and prolonged heart rate-corrected QT (PQTc, presenting at least a quarter of stroke patients) (5) intervals on ECG are frequently detected, and also associated with long-term prognosis (6, 7) in these patients.

However, in response to the extensive and sensitive capability of cardiac biomarkers to reflect an overall alteration of the brain–heart axis during AIS (8, 9), beyond indicating cardiac comorbidities (10), it is still challenging to apply the results for appropriate risk stratification (11, 12). ET indicates myocardial injury but does not define the cause of the injury from coronary artery disease (CAD) or non-CAD types of cardiac disease and even non-cardiac conditions (13). Likewise, PQTc interval indicates delayed ventricular repolarization and, in turn, risk of ventricular arrhythmias leading to sudden cardiac death (14) and future adverse outcomes in the general population (15), or those with various cardiac diseases (16–18), but can be affected by various medical conditions, even including drugs (19). Furthermore, in the setting of AIS, changes in the cardiovascular system affected by neurogenic stress (i.e., alteration of brain-heart axis via disturbances of catecholamine homeostasis and systematic inflammatory response) (20) can also lead to ET, PQTc interval, or both.

Therefore, we aimed to evaluate whether combining ET and PQTc interval can better assess the prognosis of long-term mortality, primarily focusing on the risk stratification power for cause-specific mortality in patients with AIS.

## Methods

### Study population

Prospectively registered data on patients with AIS admitted to Asan Medical Center between May 2007 and December 2011 within 24 h of symptom onset were analyzed. All patients underwent routine cardiac testing, including cardiac troponin *I* and creatine kinase MB isoenzyme (CK-MB). Additionally, 12-lead ECG investigations were performed upon admission according to the stroke protocols of our center, which abide by the 2007 guidelines (21). Patients underwent additional cardiac evaluations by a cardiologist if suspected of having acute coronary syndrome (ACS). Patients were excluded if: (1) they were diagnosed with concomitant ACS (22) upon admission, (2) their brain images or ECGs were of poor quality, or (3) they had a complete bundle branch block (QRS interval >120 ms), ventricular rhythm, or pacemaker-paced rhythm. The study protocol was approved by the Institutional Review Board of Asan Medical Center, which waived the requirement for informed consent because of the registered data analysis design of the study.

### Assessment of troponin value and QT interval duration

The lower limit of serum cardiac troponin *I* detection was 0.006 ng/ml, and the calculated 99th percentile of the URL was 0.040 ng/ml (Abbott Laboratories, Abbott Park, IL, USA). Troponin values were stratified into elevated (≥0.04 ng/ml) and non-elevated, including minimally-elevated (0.039 and 0.010 ng/ml) and non-detectable (<0.010 ng/ml) levels (23), groups.

A 12-lead ECG (GE Healthcare, Waukesha, WI), with the results processed using the Marquette 12SL ECG Analysis Program, was interpreted by a cardiologist. The QT interval was defined as the duration between the earliest QRS onset to the latest T-wave offset in the 12 ECG leads. QTc intervals were stratified by tertiles for each sex based on Fridericia’s formula for calculation of the QTc interval because it is suitable for patients with tachycardia and bradycardia (24) or atrial fibrillation (AF), leading to beat-to-beat variability in the RR interval (25).

### Data acquisition

Clinical data were obtained from the registered data, including demographic characteristics, conventional risk factors, and comorbidities. Comorbidities included ischemic heart disease (IHD, defined as a history or evidence of prior IHD on admission by 12-lead ECG), AF (defined as history or proof of AF on 12-lead ECG), ventricular hypertrophy (VH, defined as a history of hypertrophic cardiomyopathy or proof of VH on 12-lead ECG), congestive heart failure (CHF, defined as a history of cardinal manifestations and treatment for heart failure), chronic kidney disease (CKD, defined as an estimated glomerular filtration rate <60 ml/min/1.73 m^2^ on admission) and active cancer (defined as cancer within six months before enrollment, any treatment for cancer within the previous six months, or recurrent or metastatic cancer) (26). Characteristics of stroke included prior history of stroke and the National Institutes of Health Stroke Scale (NIHSS) (27) score quantifying stroke severity.

### Collection of mortality data

The nationwide official data for death certificates produced by the Korean National Statistical Office are updated annually. Follow-up patient information was obtained using the national death certificate data from the Korean National Statistical Office until December 31, 2012. Deaths were classified according to the *International Classification of Diseases, Tenth Revision* (28). Causes of death were classified as vascular death, including stroke (ICD codes: I60–I69) and cardiac causes (ICD codes: I20–I25 or I30–I52), and non-vascular death, including malignancies (ICD codes: C00–C96) and other causes.

### Statistical analysis

Continuous variables were expressed as mean ± standard deviation or median [interquartile ranges (IQR)] and compared using the Student’s *t*-test and Wilcoxon rank sum test. Categorical variables were expressed as numbers (%) and compared by the chi-square and Fisher’s exact tests.

Multivariate Cox proportional hazards models were used to determine the relationship between the combination of ET and PQTc interval and long-term mortality, including all-cause and cause-specific mortality related to vascular and non-vascular death. The hazard ratio (HR) was reported with a 95% confidence interval (CI). Variables were included in a stepwise method based on previous research, with a consideration of the impact of stroke severity on changes in cardiac biomarkers as well as long-term mortality (7, 29, 30). Model 1 included adjustments for age, sex, conventional risk factors, comorbidities, and all laboratory results, and model 2 additionally included the NIHSS scores on top of the Model 1 for estimating HR. The timing of events according to the combination of ET and PQTc interval was assessed by the Kaplan–Meier method, with curves compared by log-rank tests. All reported *p*-values are two-sided, with *p* < 0.05 considered statistically significant. All statistical analyses were performed using SPSS for Windows version 17.0 (SPSS Inc., Chicago, IL, USA).

## Results

### Baseline characteristics

**Figure 1:**
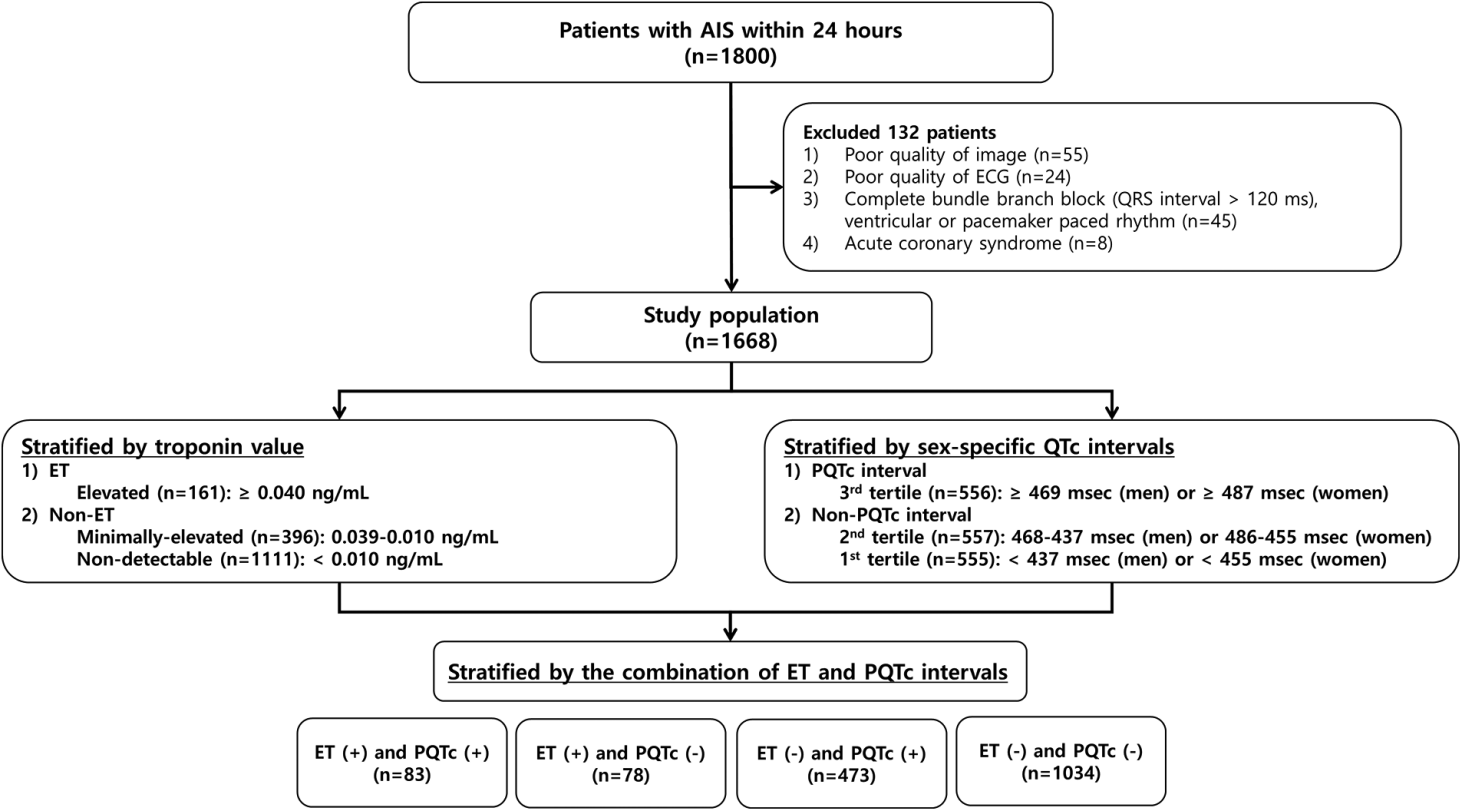
Flowchart of the patient selection process and classification by the troponin value and the QTc interval. AIS, acute ischemic stroke; ECG, electrocardiogram; ET, elevated troponin; PQTc, prolonged heart rate-corrected QT.

A total of 1,668 patients were eligible for this study, with a mean age of 66.0 ± 12.4 years (range, 24–96 years), and 1,018 (61.0%) were men. They had a mean of 0.06 ± 0.70 ng/ml of troponin value and a mean of 462.3 ± 43.9 ms (range, 343–809 ms) of QTc interval. After measuring the troponin level, 161 (9.7%) patients were assigned to the ET group. Next, according to the sex-specific QTc interval, patients with the highest tertile of QTc interval (≥469 ms in men and ≥487 ms in women) were classified into the PQTc interval group. Then, patients were stratified into four groups based on the combination of ET and PQTc intervals (Figure 1).

Of the characteristics, patients with ET, PQTc interval, or both were older and had a higher prevalence of comorbidities, including AF, VH, IHD, CHF, CKD, active cancer, and a higher NIHSS score than those with neither ET nor PQTc interval. They also had higher leukocyte and lower platelet and hemoglobin counts, lower albumin, and higher C-reactive protein concentrations than patients with neither ET nor PQTc interval (Table 1).

**Table 1:** Patient characteristics according to the combination of ET and PQTc interval.

Variable	Stratified by the combination of ET and PQTc interval				*p-*value^a^
	ET (*N* = 161)		Non-ET (*N* = 1507)		
	PQTc (*n* = 83)	Non-PQTc (*n* = 78)	PQTc (*n* = 473)	Non-PQTc (*n* = 1034)	
Age (years)	69.6 ± 13.6	67.6 ± 12.4	66.5 ± 12.2	65.4 ± 12.4	0.01
Male	49 (59.0)	45 (57.7)	290 (61.3)	634 (61.3)	0.91
Conventional risk factors					
Hypertension	58 (69.9)	52 (66.7)	319 (67.4)	632 (61.1)	0.06
Diabetes mellitus	17 (20.5)	19 (24.4)	129 (27.3)	252 (24.4)	0.49
Hyperlipidemia	17 (20.5)	14 (17.9)	106 (22.4)	241 (23.3)	0.69
Current smoking	19 (22.9)	25 (32.1)	143 (30.2)	327 (31.6)	0.41
Comorbidities					
AF	44 (53.0)	25 (32.1)	166 (35.1)	237 (22.9)	<0.01
VH	33 (39.8)	34 (43.6)	131 (27.7)	231 (22.3)	<0.01
IHD	22 (26.5)	15 (19.2)	60 (12.7)	132 (12.8)	<0.01
CHF	23 (27.7)	11 (14.1)	66 (14.0)	73 (7.1)	<0.01
CKD	23 (27.7)	10 (12.8)	73 (15.4)	117 (11.3)	<0.01
Active cancer	8 (9.6)	17 (21.8)	17 (3.6)	46 (4.4)	<0.01
Characteristics of stroke					
Previous stroke	21 (25.3)	21 (26.9)	132 (27.9)	259 (25.0)	0.70
NIHSS score	11 [3, 15]	5 [2, 10.25]	5 [2, 11]	4 [2, 8]	<0.01
Laboratory results					
White blood cell (10^3^/ul)	9.1 ± 3.3	8.1 ± 2.9	8.7 ± 3.2	7.9 ± 2.7	<0.01
Platelet (10^3^/ul)	216.2 ± 88.7	196.7 ± 85.6	226.0 ± 72.2	223.0 ± 61.2	0.01
Hemoglobin (g/dl)	13.1 ± 2.0	13.2 ± 2.1	14.0 ± 2.1	13.9 ± 1.8	<0.01
Glucose (mg/dl)	150.3 ± 60.0	137.6 ± 43.2	149.6 ± 52.9	143.7 ± 58.8	0.16
Low-density lipoprotein (mg/dl)	107.3 ± 38.9	106.0 ± 36.2	105.9 ± 33.3	109.8 ± 34.5	0.25
High-density lipoprotein (mg/dl)	43.6 ± 11.5	43.0 ± 12.5	43.3 ± 11.9	42.9 ± 11.9	0.91
Albumin (g/dl)	3.6 ± 0.5	3.7 ± 0.5	3.8 ± 0.5	3.8 ± 0.4	<0.01
Homocysteine (mmol/ml)	15.0 ± 6.1	14.8 ± 5.5	14.5 ± 5.8	14.9 ± 7.7	0.81
C-reactive protein (mg/dl)	1.1 ± 2.4	1.2 ± 2.6	0.9 ± 2.5	0.6 ± 1.8	0.02

Variables are presented as mean ± SD, median [interquartile range], or number (%).

AF, atrial fibrillation; CHF, congestive heart failure; CKD, chronic kidney disease; ET, elevated troponin; IHD, ischemic heart disease; NIHSS, National Institutes of Health Stroke Scale; PQTc, prolonged heart rate-corrected QT; VH, ventricular hypertrophy.

^a^ *p*-values are calculated by Pearson chi-square test, Fisher’s exact test, ANOVA test, and Kruskal–Wallis test as appropriate**.**

### Prognostic significance of combined ET and PQTc interval

Over a median follow-up period of 33 months (IQR, 20–48 months), 323 (19.4%) patients died, including vascular death (*n* = 190) and non-vascular death (*n* = 133).

**Figure 2:**
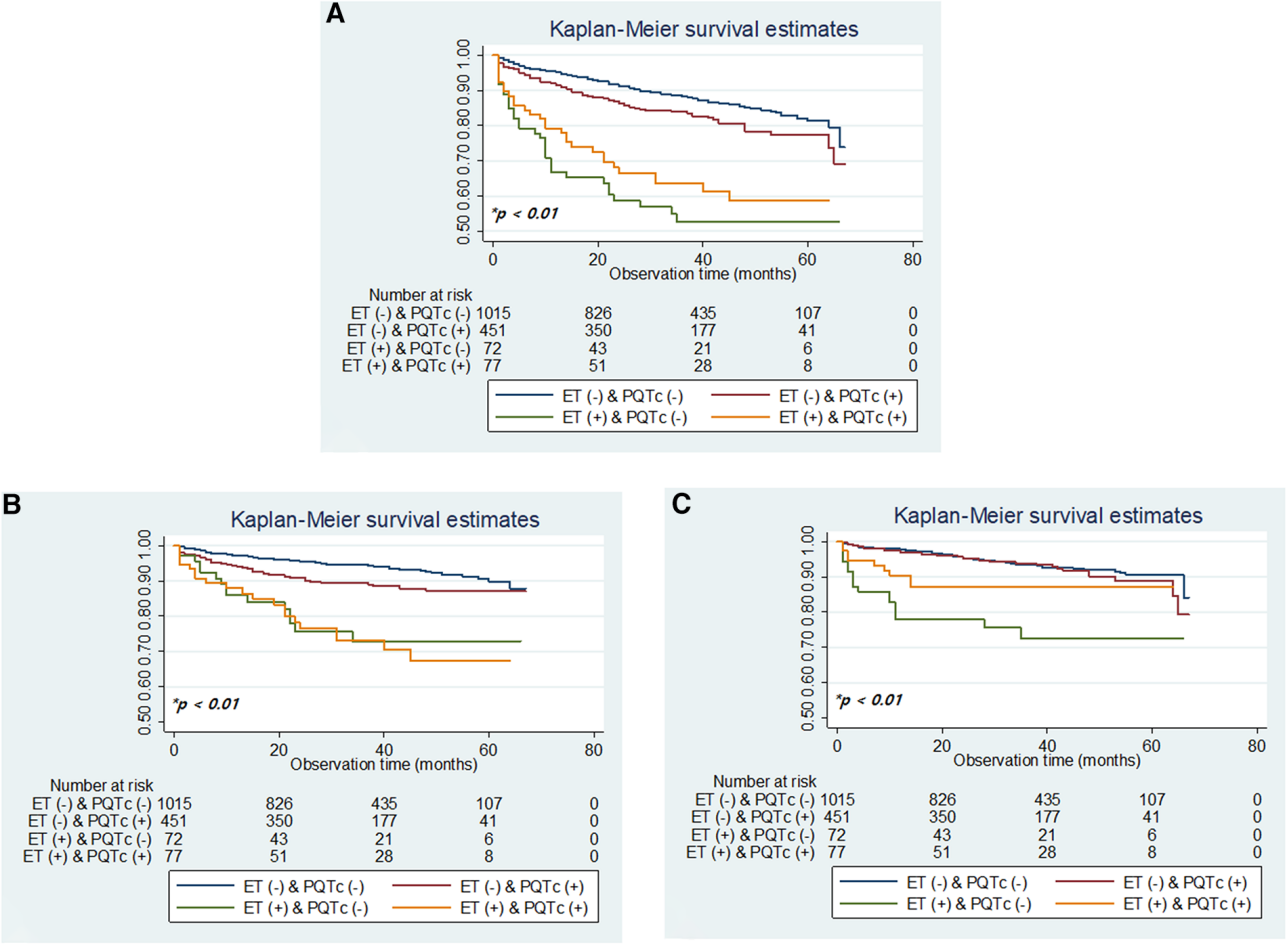
Kaplan–meier plots of overall survival (**A**) and survival by vascular death and non-vascular death (**B,C**) according to the combination of ET and PQTc interval. ET, elevated troponin; PQTc, prolonged heart rate-corrected QT. *p-values determined using the log-rank test.

Kaplan–Meier analysis of long-term survival revealed that the risk of all-cause and cause-specific mortality increased in patients with ET, in combination with PQTc interval. Overall, patients with ET alone showed the highest all-cause and non-vascular mortality, followed by patients with concurrent ET and PQTc, showing the 2nd highest all-cause mortality and the highest vascular mortality, and those with PQTc alone (Figure 2).

Adjusted multivariable analysis using the Cox-regression model with stepwise selection of clinically relevant variables indicated that ET (HR: 4.38, 95% CI: 2.94–6.53) or PQTc interval (HR: 1.53, 95% CI: 1.16–2.01) alone or together (HR: 1.77, 95% CI: 1.16–2.71) was significantly associated with increased risk of all-cause mortality in the model 1, and predictive value of ET alone (HR: 4.05, 95% CI: 2.72–6.04) was still preserved even after additional adjusting for stroke severity on top of the model 1 (Table 2).

**Table 2:** Incidence rate per 1,000 person-months and unadjusted and adjusted hazard ratios for the combination of ET and PQTc interval to predict clinical outcomes during the 6-year follow-up period.

Quartiles	Number of events	Incidence, %	Unadjusted		Adjusted^a^		Adjusted^b^	
			HR	95% CI	HR	95% CI	HR	95% CI
All-cause mortality								
ET (−) and PQTc (−)	149/1034 (14.4)	4.0	Reference		Reference		Reference	
ET (−) and PQTc (+)	101/473 (21.4)	6.6	1.59	1.23–2.04	1.53	1.16–2.01	1.30	0.98–1.72
ET (+) and PQTc (−)	38/78 (46.6)	19.6	4.39	3.07–6.27	4.38	2.94–6.53	4.05	2.72–6.04
ET (+) and PQTc (+)	35/83 (42.2)	14.5	3.47	2.40–5.02	1.77	1.16–2.71	1.23	0.80–1.89
C-index			0.624		0.819		0.854	
Vascular death								
ET (−) and PQTc (−)	79/1034 (7.6)	2.1	Reference		Reference		Reference	
ET (−) and PQTc (+)	66/473 (14.0)	4.3	1.95	1.40–2.70	1.76	1.23–2.51	1.42	0.98–2.06
ET (+) and PQTc (−)	19/78 (24.4)	9.8	4.05	2.45–6.68	4.16	2.40–7.22	3.88	2.26–6.68
ET (+) and PQTc (+)	26/83 (31.3)	10.8	4.77	3.06–7.43	2.41	1.45–4.03	1.58	0.94–2.68
C-index			0.639		0.812		0.859	
Non-vascular death								
ET (−) and PQTc (−)	70/1034 (6.8)	1.9	Reference		Reference		Reference	
ET (−) and PQTc (+)	35/473 (7.4)	2.3	1.18	0.79–1.77	1.33	0.85–2.10	1.27	0.81–2.01
ET (+) and PQTc (−)	19/78 (24.4)	9.8	4.83	2.91–8.02	3.95	2.08–7.51	3.76	1.97–7.15
ET (+) and PQTc (+)	9/83 (10.8)	3.7	1.95	0.98–3.91	0.91	0.41–2.04	0.78	0.35–1.77
C-index			0.599		0.902		0.907	

CI, confidence interval; ET, elevated troponin; HR, hazard ratio; NIHSS, National Institutes of Health Stroke Scale; PQTc, prolonged heart rate-corrected QT.

^a^ Model 1, adjusted for age, sex, conventional risk factors, six comorbidities, and all laboratory results in Table 1.

^b^ Model 2, adjusted for model 1 plus NIHSS score.

In the cause-specific mortality analysis, patients with ET (HR: 4.16, 95% CI: 2.40–7.22) or PQTc interval (HR: 1.76, 95% CI: 1.23–2.51) alone or both (HR: 2.41, 95% CI: 1.45–4.03) had a higher risk of vascular death, whereas patients with ET (HR: 3.95, 95% CI: 2.08–7.51) alone had a higher risk of non-vascular death, than those neither ET nor PQTc interval. In addition, the predictive value of ET alone for vascular death (HR: 3.88, 95% CI: 2.26–6.68) and non-vascular death (HR: 3.76, 95% CI: 1.97–7.15) was still preserved even after additional adjusting for stroke severity on top of the model 1 (Table 2).

### Overall burden according to the troponin value and QTc interval

For the reappraisal of the prognostic significance of combining ET and PQTc interval, a substudy based on the three by three subgroups according to the troponin value and QTc interval was done to verify the dose-response relationship to the comorbidity burden and stroke severity as evidence of the causality.

**Figure 3:**
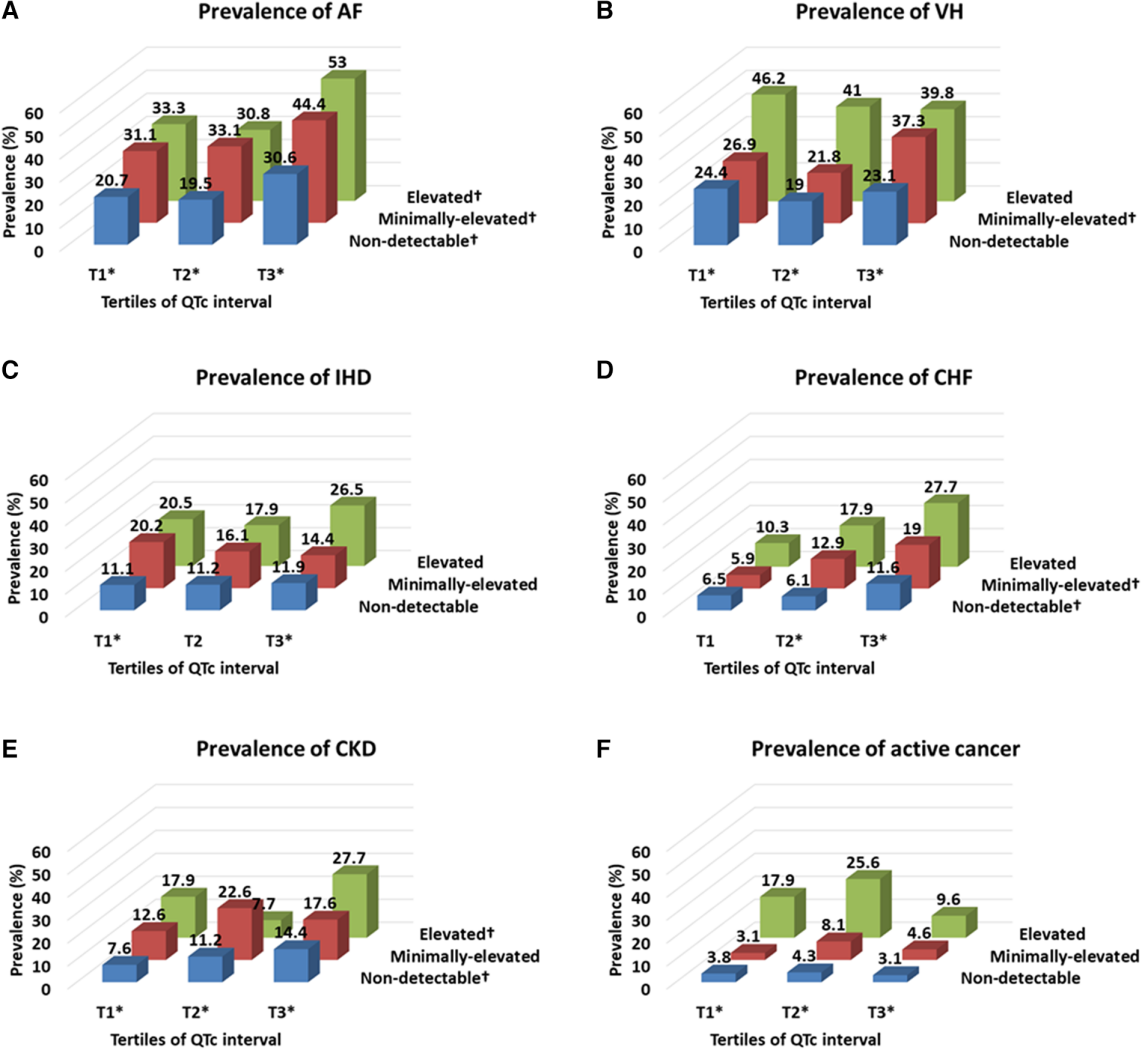
Prevalence of comorbidities according to the troponin level and QTc interval. Troponin level was classified by the elevated (≥0.040 ng/ml), minimally-elevated (0.039–0.010 ng/ml) and non-detectable (<0.010 ng/ml) levels, and QTc-interval was classified by the tertile of QTc interval in each sex (cut-off points: 437 and 469 ms for men and 455 and 487 ms for women). AF, atrial fibrillation; CHF, congestive heart failure; CKD, chronic kidney disease; IHD, ischemic heart disease; VH, ventricular hypertrophy. *p-value <0.05 by Pearson chi-square test according to the categorized troponin value in each categorized QTc interval group. †*p*-value <0.05 by Pearson chi-square test according to the categorized QTc interval in each categorized troponin value group.

**Figure 4:**
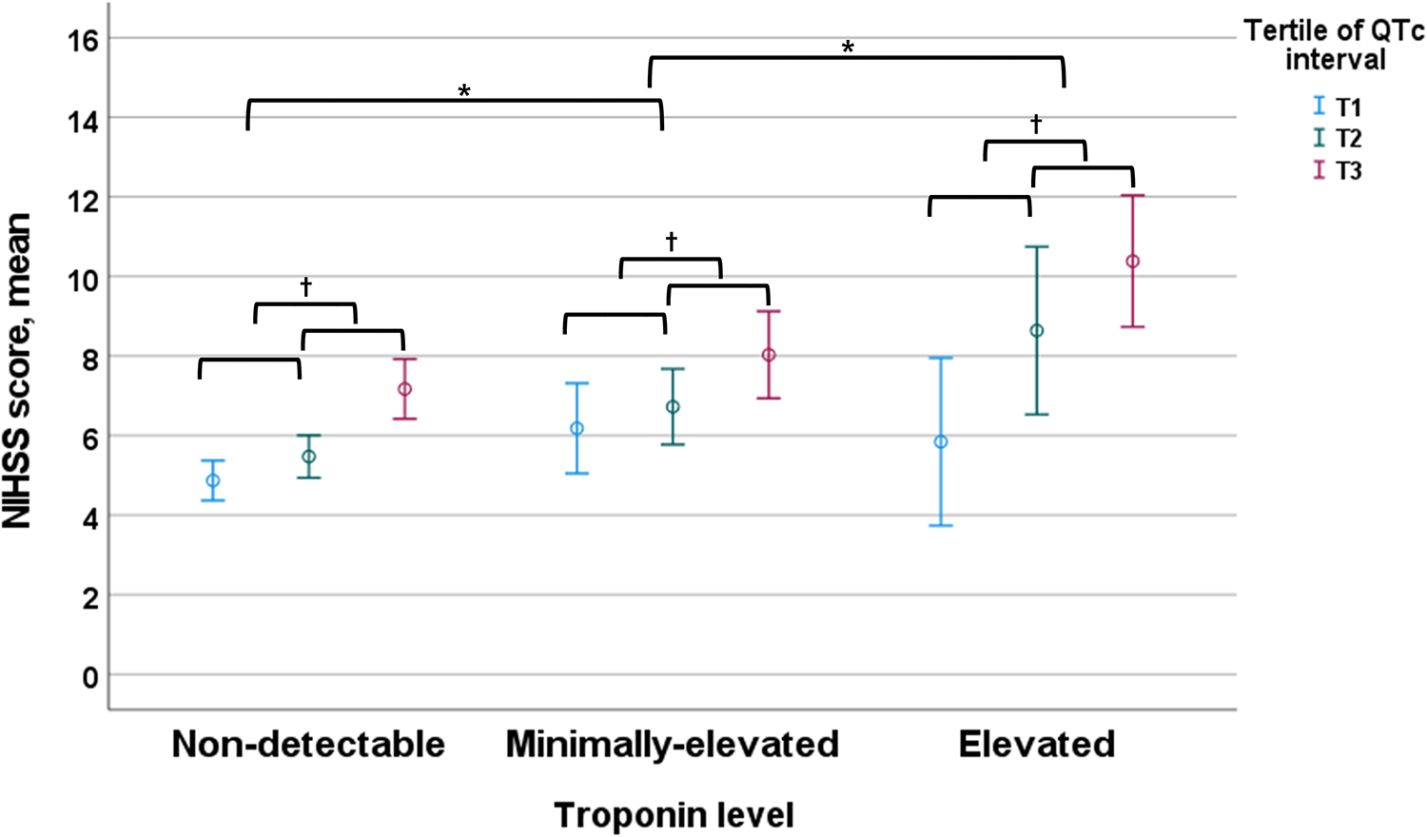
NIHSS score according to the troponin level and QTc interval. Troponin level was classified by the elevated (≥0.040 ng/ml), minimally-elevated (0.039–0.010 ng/ml) and non-detectable (<0.010 ng/ml) levels, and QTc-interval was classified by the tertile of QTc interval in each sex (cut-off points: 437 and 469 ms for men and 455 and 487 ms for women). NIHSS, National Institutes of Health Stroke Scale. *p-value <0.05 by ANOVA with Duncan post-hoc test according to the categorized troponin value group. ^†^*p*-value <0.05 by ANOVA with Duncan post-hoc test according to the categorized QTc interval in each categorized troponin value group.

The overall prevalence of comorbidities, especially AF and CHF, gradually increased with the elevation of troponin values and further increased by prolonging QTc intervals. In contrast, the prevalence of VH, IHD, CKD, and active cancer gradually increased with the elevation of troponin value regardless of QTc interval (Figure 3). In addition, the mean NIHSS score gradually increased with the elevation of troponin value and increased further with the prolongation of QTc intervals (Figure 4).

**Figure 5:**
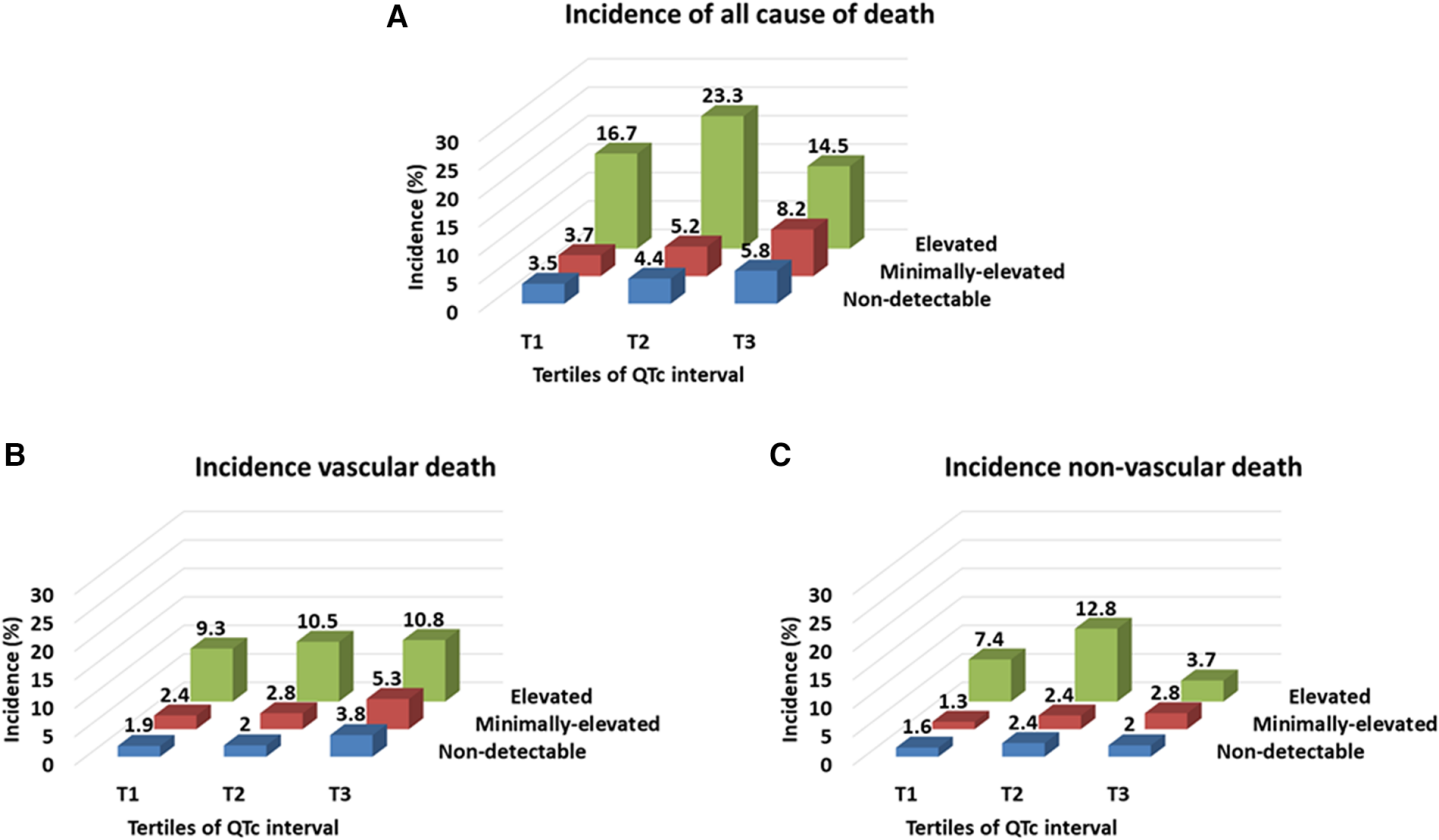
Incidence rate per 1,000 person-months of all-cause mortality (**A**), and cause-specific mortality related to vascular and non-vascular death (**B,C**) according to the troponin level and QTc interval. Troponin level was classified by the elevated (≥0.040 ng/ml), minimally-elevated (0.039–0.010 ng/ml) and non-detectable (<0.010 ng/ml) levels, and QTc-interval was classified by the tertile of QTc interval in each sex (cut-off points: 437 and 469 ms for men and 455 and 487 ms for women).

Finally, the incidence of all-cause and vascular death increased gradually with the elevation of troponin value and further increased with the prolongation of QTc intervals. In contrast, the incidence of non-vascular death was the highest in patients with ET alone without PQTc interval (Figure 5).

## Discussion

### Summary of the main findings

This study reveals the prognostic significance of ET and PQTc interval on long-term mortality after stroke, which improved, especially when combined, for risk stratification to predict all-cause and cause-specific mortality (Figure 2 and Table 2), compared to the results of dichotomized ET or PQTc (Supplementary Table S1, S2 and Figure S1). Furthermore, comorbidity burden, especially AF and CHF, and stroke severity gradually increased both with troponin value and QTc-interval (Figures 3, 4). Thus, as the issues mentioned above, due to the extensive and sensitive capability of cardiac biomarkers to reflect an overall alteration of the brain–heart axis during AIS, combining ET and PQTc interval enhances risk stratification for long-term mortality while facilitating the discerning ability for the burden of comorbidities and stroke severity. For these reasons, ET, PQTc interval alone or both can be helpful in predicting future vascular death, whereas ET alone can be helpful in predicting future non-vascular death (Figure 5).

### Risk stratification value of combined ET and PQTc interval

In our study, ET or PQTc alone, or together was closely associated with all-cause and vascular death after adjusting for clinically-relevant variables, otherwise their prognostic significance somewhat diminished after additional adjustment for stroke severity, particularly in patients with ET and concurrent PQTc interval (Table 2). This finding indicates the close and commonly shared interaction of these cardiac biomarkers to the stroke severity in patients with AIS, as mentioned in a previous study (29). Furthermore, we still reconfirmed their close correlation in the current substudy. At first, a combined analysis of three by three subgroups according to the troponin value and QTc interval indicated a more precise relationship between cardiac biomarkers and underlying conditions, including cardiac comorbidities (i.e., in particular, AF and CHF) and neurological statuses (i.e., stroke severity measured by the NIHSS score), in a dose-responsive manner (Figures 3, 4). In addition, we also recognized a strong interaction within these biomarkers because there is a gradually increasing trend of prolonging QTc interval according to increasing troponin value in a dose-dependent manner (Supplementary Figure S2). Thus, ET combined with PQTc interval can be regarded as a specialized indicator for high-risk subjects, accompanying high-risk cardiogenic embolic sources (31), which, in turn, leads to more extensive stroke attributed to a cardiogenic embolism (32, 33).

### Risk stratification value of ET and PQTc interval alone

Interestingly, regardless of PQTc interval, ET alone showed the most robust predictive value for all-cause and cause-specific mortality, including both vascular and non-vascular death, even after adjusting for stroke severity on top of the clinically-relevant variables (Table 2). This finding indicates the capability of serum troponin value, reflecting a wide range of comorbidity burdens in line with previous studies (9), and this study. In a combined analysis of three by three subgroups according to the troponin value and QTc interval, the prevalence of comorbidities, including AF, CHF, LVH, IHD, CKD, and active cancer, gradually increased with increasing troponin in a dose-responsive manner, regardless of QTc interval (Figure 3). Therefore, in contrast with the value of ET combined with PQTc interval as a specialized indicator for vascular burden primarily reflecting the change of brain–heart axis in AIS patients, ET alone without PQTc interval remarks overall comorbidity burden, particularly in a relatively low-stress situation, such as a minor stroke accompanying less severe neurological deficits (9). For this reason, ET alone shows a good performance in predicting non-vascular death, primarily due to cancer or cancer-related stroke, which is notable (Figure 5) (34, 35).

Likewise, the clinical value of QTc interval was also further improved with a combination of troponin values in patients with AIS. In a separate analysis, the prognostic value of dichotomized PQTc interval showed a limited clinical value because it's statistical significance was completely diminished after additional adjusting stroke severity (Supplementary Table 2). However, PQTc alone without ET still tended to be associated with all-cause and vascular death, even after adjusting for stroke severity on top of the clinically relevant variables (Table 2). Thus, likewise, ET without PQTc interval, PQTc interval alone without ET also remarks comorbidity burden primarily in high-risk cardiac conditions, such as AF and CHF, especially in the setting of a minor stroke, as mentioned.

### Issues of generalizability

In this cohort, the overall incidence of ET was 9.7% which was in line with a previous systematic review showing a 7.8%–33% incidence of ET across heterogeneous cohorts with AIS (4). On the contrary, this cohort's proportion of the PQTc interval was not comparable with previous studies due to different cut-off values and calculating formulas for defining the PQTc interval. We used a tertile for defining the PQTc interval instead of the generally adopted cut-off value (i.e., QTc interval >440–450 ms in men and >460 ms in women) and also used the Fridericia formula for calculating QTc interval instead of the Bazett formula as following reasons.

Until now, there has been no consensus on a fair cut-off value to define the clinically-significant PQTc interval, especially for patients with AIS. Traditionally, an abnormal range of QTc values was defined as more prolonged than the 97.5th percentile among the general population, conforming to a Gaussian distribution of QTc interval (36). The community-based definition of PQTc interval shows a good performance in predicting all-cause and coronary heart disease mortality, particularly in a healthy population, even in a meta-analysis (37). However, in the present study targeting patients with AIS, the mean QTc interval (462.3 ± 43.9 ms) was almost 50 ms longer than in normal populations. Thus, we previously proposed that an appropriate cut-off value defining PQTc interval in stroke patients should be higher than the standard range to improve its practical value, considering many factors contributing to PQTc intervals in these patients, such as older age, more burden of comorbidities (i.e., cardiac disease and drugs), and neurological status (i.e., autonomic dysregulation induced by neurogenic stress) (7).

Furthermore, in terms of an algorithm to calculate the QTc normalized to a heart rate of 60 beats/min (24), the Bazett formula, regarded as a standard method, has a limitation to its usage, including over-estimating the QTc interval at high heart rates and in the setting of the instantaneous preceding RR interval or the interval between beats on ECG (25). In this sense, AF is frequently associated with rapid heart rates and irregular RR intervals, which often prolong the QT interval and cause QTc estimation to become highly variable even at the average heart rate (38). Consequently, QTc measurement has been revisited recently, using the Fridericia formula instead of the Bazett to improve its accuracy in patients with AF (39). Therefore, with consideration of the vital importance of AF in ischemic stroke patients as a prevalent arrhythmia, a significant source of embolic stroke, and a major determinant of outcomes (40), reassessments of the QTc interval with the Fridericia formula could be more helpful for improving the clinical value of QTc interval for risk stratification.

## Study limitations

This study had several limitations. First, this study was performed in a single center, which may limit the generalizability of our results. Furthermore, we could not show the prognostic value for non-fatal long-term outcomes, such as major adverse cerebro-cardiovascular events (MACCE), because patient follow-up information was obtained using the national death certificate data from the Korean National Statistical Office. In addition, as our study covers a 5-year follow-up period, we have a limitation of a lack of temporal trend analysis, considering possible bias that more recently included AIS patients might have benefited from improved therapeutic approaches and post-stroke care. However, we initially selected prospectively registered data between 2007 and 2011 because all consecutive AIS patients underwent routine cardiac testing upon admission according to the protocols, abide by the 2007 guidelines (21). Furthermore, in this period, the strategy for managing AIS focused on intravenous thrombolysis until endovascular treatment's proven efficacy and safety, which was initially mentioned as a complementary treatment in the 2013 guideline (41) and confirmed as the primary treatment in the 2015 guideline (42). Therefore, issues of variation of patients or different treatment protocols may be lessened for this enrollment period. Second, the precise incidence of ET and PQTc interval remains tentative because serum cardiac enzyme and ECG were performed at single time points rather than over time. Furthermore, the definition of comorbidities was primarily based on the historical information or ECG results upon admission, thus, a risk of underestimation of subclinical conditions remains. In addition, based on the single measurement of cardiac biomarkers, we still have a risk of underestimating concomitant ACS or stress-induced cardiomyopathy, which should be diagnosed by serial measurement of serum troponin with serial ECG tests to confirm the dynamic pattern compatible with the ACS (22) or repeated echocardiography to show the compatible structural abnormalities (i.e., reversible apical ballooning) (43). To overcome these problems, we are conducting a prospective trial with serial measurements of troponin and ECG in patients with AIS (Clinical implications of elevated cardiac troponin-*I* elevation in acute stroke patients; KCT0000682; https://cris.nih.go.kr/cris). This study aims to reveal serial changes in serum troponin values and QTc intervals and their association with MACCE in these patients (6). Finally**,** the cut-off value for the dichotomized PQTc interval according to the highest tertile of sex-specific QTc interval is still arbitrary. Thus, in a separate analysi, we investigated an appropriate cut-off value for defining PQTc interval considering sensitivity and specificity. Finally, the cut-off value of QTc interval at 458.65 ms showed the highest sensitivity (0.506) and specificity (0.576), and QTc interval at 444.93 ms showed the highest Youden J value (0.187) in male patients. In addition, the cut-off value of QTc interval at 470.83 ms showed the highest sensitivity (0.537) and specificity (0.535), and QTc-interval at 530.76 ms showed the highest Youden J value (0.101) in female patients (Supplemental Figure S3). Therefore, a future study is needed to identify an appropriate cut-off value for defining PQTc interval in AIS patients, especially based on the serially measured QTc interval, considering the dynamic change of QTc interval during the acute stage of stroke. Furthermore, various medications that affect QTc interval should be adjusted, considering the dosage and duration of treatment. However, the effect of various medications on QTc interval did not differ significantly across our study’s tertiles of QTc intervals.

## Conclusions

In patients with AIS, cardiac biomarkers, including ET and PQTc interval, were associated with an increased risk of long-term mortality, parallel with the increasing prevalence of cardiovascular risk profile and stroke severity according to the troponin value and QTc interval. Furthermore, the combination of troponin value and QTc interval may enhance risk assessment to adjust for the influence of stroke severity on long-term mortality while facilitating appropriate reappraisal of cause-specific mortality in patients with AIS.

## Data Availability

The datasets presented in this article are not readily available. Requests to access the datasets should be directed to caesar-ahn@hanmail.net.

## References

[1] Global, regional, and national burden of stroke and its risk factors, 1990–2019: a systematic analysis for the global burden of disease study 2019. Lancet Neurol. (2021) 20(10):795–820. 10.1016/S1474-4422(21)00252-0 34487721 PMC8443449

[2] Kumar S Selim MH Caplan LR. Medical complications after stroke. Lancet Neurol. (2010) 9(1):105–18. 10.1016/S1474-4422(09)70266-2 20083041

[3] Powers WJ Rabinstein AA Ackerson T Adeoye OM Bambakidis NC Becker K Guidelines for the early management of patients with acute ischemic stroke: 2019 update to the 2018 guidelines for the early management of acute ischemic stroke: a guideline for healthcare professionals from the American heart association/American stroke association. Stroke. (2019) 50(12):e344–418. 10.1161/STR.0000000000000211 31662037

[4] Kerr G Ray G Wu O Stott DJ Langhorne P. Elevated troponin after stroke: a systematic review. Cerebrovasc Dis. (2009) 28(3):220–6. 10.1159/000226773 19571535

[5] Khechinashvili G Asplund K. Electrocardiographic changes in patients with acute stroke: a systematic review. Cerebrovasc Dis. (2002) 14(2):67–76. 10.1159/000064733 12187009

[6] Ahn SH Kim YH Lee JS Han JH Kim SY Kang DW Troponin I levels and long-term outcomes in acute ischemic stroke patients. J Am Coll Cardiol. (2019) 73(4):525–6. 10.1016/j.jacc.2018.11.022 30704584

[7] Ahn S-H Lee J-S Kim Y-H Yun M-S Han J-H Kim S-Y Prognostic significance of prolonged corrected QT interval in acute ischemic stroke. Front Neurol. (2021) 12(2319). 10.3389/fneur.2021.759822 PMC8720760 34987464

[8] Sandau KE Funk M Auerbach A Barsness GW Blum K Cvach M Update to practice standards for electrocardiographic monitoring in hospital settings: a scientific statement from the American heart association. Circulation. (2017) 136(19):e273–344. 10.1161/CIR.0000000000000527 28974521

[9] Eggers KM Jernberg T Lindahl B. Cardiac troponin elevation in patients without a specific diagnosis. J Am Coll Cardiol. (2019) 73(1):1–9. 10.1016/j.jacc.2018.09.082 30621937

[10] Xu C Zheng A He T Cao Z. Brain-heart axis and biomarkers of cardiac damage and dysfunction after stroke: a systematic review and meta-analysis. Int J Mol Sci. (2020) 21(7). 10.3390/ijms21072347 PMC7178236 32231119

[11] Twerenbold R Jaffe A Reichlin T Reiter M Mueller C. High-sensitive troponin T measurements: what do we gain and what are the challenges? Eur Heart J. (2012) 33(5):579–86. 10.1093/eurheartj/ehr492 22267244

[12] Priori SG Blomstrom-Lundqvist C Mazzanti A Blom N Borggrefe M Camm J Elliott PM Fitzsimons D Hatala R Hindricks G Kirchhof P Kjeldsen K Kuck KH Hernandez-Madrid A Nikolaou N Norekval TM Spaulding C Van Veldhuisen DJ. 2015 ESC guidelines for the management of patients with ventricular arrhythmias and the prevention of sudden cardiac death: the task force for the management of patients with ventricular arrhythmias and the prevention of sudden cardiac death of the European society of cardiology (ESC). endorsed by: association for European paediatric and congenital cardiology (AEPC). Eur Heart J. (2015) 36(41):2793–867. 10.1093/eurheartj/ehv316 26320108

[13] Mahajan VS Jarolim P. How to interpret elevated cardiac troponin levels. Circulation. (2011) 124(21):2350–4. 10.1161/CIRCULATIONAHA.111.023697 22105197

[14] Algra A Tijssen JG Roelandt JR Pool J Lubsen J. QTc prolongation measured by standard 12-lead electrocardiography is an independent risk factor for sudden death due to cardiac arrest. Circulation. (1991) 83(6):1888–94. 10.1161/01.CIR.83.6.1888 2040041

[15] Schouten EG Dekker JM Meppelink P Kok FJ Vandenbroucke JP Pool J. QT interval prolongation predicts cardiovascular mortality in an apparently healthy population. Circulation. (1991) 84(4):1516–23. 10.1161/01.CIR.84.4.1516 1914093

[16] Reusser A Blum S Aeschbacher S Eggimann L Ammann P Erne P QTc interval, cardiovascular events and mortality in patients with atrial fibrillation. Int J Cardiol. (2018) 252:101–5. 10.1016/j.ijcard.2017.11.078 29203211

[17] Ishikawa J Ishikawa S Kario K. Prolonged corrected QT interval is predictive of future stroke events even in subjects without ECG-diagnosed left ventricular hypertrophy. Hypertension. (2015) 65(3):554–60. 10.1161/HYPERTENSIONAHA.114.04722 25534703

[18] Hasanien AA Drew BJ Howie-Esquivel J. Prevalence and prognostic significance of long QT interval in patients with acute coronary syndrome: review of the literature. J Cardiovasc Nurs. (2014) 29(3):271–9. 10.1097/JCN.0b013e31829bcf1a 23839573

[19] Morita H Wu J Zipes DP. The QT syndromes: long and short. Lancet. (2008) 372(9640):750–63. 10.1016/S0140-6736(08)61307-0 18761222

[20] Palma JA Benarroch EE. Neural control of the heart: recent concepts and clinical correlations. Neurology. (2014) 83(3):261–71. 10.1212/WNL.0000000000000605 24928126

[21] Adams HP Jr del Zoppo G Alberts MJ Bhatt DL Brass L Furlan A Guidelines for the early management of adults with ischemic stroke: a guideline from the American heart association/American stroke association stroke council, clinical cardiology council, cardiovascular radiology and intervention council, and the atherosclerotic peripheral vascular disease and quality of care outcomes in research interdisciplinary working groups: the American academy of neurology affirms the value of this guideline as an educational tool for neurologists. Circulation. (2007) 115(20):e478–534. 10.1161/CIRCULATIONAHA.107.181486 17515473

[22] Thygesen K Alpert JS Jaffe AS Chaitman BR Bax JJ Morrow DA Fourth universal definition of myocardial infarction (2018). J Am Coll Cardiol. (2018) 18:36941–9. 10.1016/j.jacc.2018.08.1038 30153967

[23] van den Bos EJ Constantinescu AA van Domburg RT Akin S Jordaens LJ Kofflard MJ. Minor elevations in troponin I are associated with mortality and adverse cardiac events in patients with atrial fibrillation. Eur Heart J. (2011) 32(5):611–7. 10.1093/eurheartj/ehq491 21252170

[24] Rautaharju PM Surawicz B Gettes LS Bailey JJ Childers R Deal BJ AHA/ACCF/HRS recommendations for the standardization and interpretation of the electrocardiogram: part IV: the ST segment, T and U waves, and the QT interval: a scientific statement from the American heart association electrocardiography and arrhythmias committee, council on clinical cardiology; the American college of cardiology foundation; and the heart rhythm society. Endorsed by the international society for computerized electrocardiology. J Am Coll Cardiol. (2009) 53(11):982–91. 10.1016/j.jacc.2008.12.014 19281931

[25] Dash A Torado C Paw N Fan D Pezeshkian N Srivatsa U. QT Correction in atrial fibrillation – measurement revisited. J Electrocardiol. (2019) 56:70–6. 10.1016/j.jelectrocard.2019.06.009 31325620

[26] Lee AY Levine MN Baker RI Bowden C Kakkar AK Prins M Low-molecular-weight heparin versus a coumarin for the prevention of recurrent venous thromboembolism in patients with cancer. N Engl J Med. (2003) 349(2):146–53. 10.1056/NEJMoa025313 12853587

[27] Brott T Adams HP Jr Olinger CP Marler JR Barsan WG Biller J Measurements of acute cerebral infarction: a clinical examination scale. Stroke. (1989) 20(7):864–70. 10.1161/01.STR.20.7.864 2749846

[28] Organization WH. International statistical classification of diseases and related health problems. Tenth revision. Vol. 1: Tabular list (1992); vol. 2: instruction manual (1993); vol. 3: index (1994). Geneva: WHO (1992). 1992.

[29] Ahn SH Kim YH Shin CH Lee JS Kim BJ Kim YJ Cardiac vulnerability to cerebrogenic stress as a possible cause of troponin elevation in stroke. J Am Heart Assoc. (2016) 5(10):e004135. 10.1161/jaha.116.004135 27792642 PMC5121511

[30] Ahn SH Lee JS Kim YH Kim BJ Kim YJ Kang DW Prognostic significance of troponin elevation for long-term mortality after ischemic stroke. J Stroke. (2017) 19(3):312–22. 10.5853/jos.2016.01942 28877565 PMC5647632

[31] Haeusler KG Laufs U Endres M. Chronic heart failure and ischemic stroke. Stroke. (2011) 42(10):2977–82. 10.1161/STROKEAHA.111.628479 21903953

[32] Hijazi Z Siegbahn A Andersson U Granger CB Alexander JH Atar D High-sensitivity troponin I for risk assessment in patients with atrial fibrillation: insights from the apixaban for reduction in stroke and other thromboembolic events in atrial fibrillation (ARISTOTLE) trial. Circulation. (2014) 129(6):625–34. 10.1161/CIRCULATIONAHA.113.006286 24226808

[33] Aimo A Januzzi JL Vergaro G Ripoli A Latini R Masson S Prognostic value of high-sensitivity troponin T in chronic heart failure. Circulation. (2018) 137(3):286–97. 10.1161/CIRCULATIONAHA.117.031560 29335288

[34] Cardinale D Sandri MT Colombo A Colombo N Boeri M Lamantia G Prognostic value of troponin I in cardiac risk stratification of cancer patients undergoing high-dose chemotherapy. Circulation. (2004) 109(22):2749–54. 10.1161/01.CIR.0000130926.51766.CC 15148277

[35] Alexandre J Cautela J Ederhy S Damaj GL Salem JE Barlesi F Cardiovascular toxicity related to cancer treatment: a pragmatic approach to the American and European cardio-oncology guidelines. J Am Heart Assoc. (2020) 9(18):e018403. 10.1161/JAHA.120.018403 32893704 PMC7727003

[36] Rautaharju PM Prineas RJ Kadish A Larson JC Hsia J Lund B. Normal standards for QT and QT subintervals derived from a large ethnically diverse population of women aged 50 to 79 years [the women’s health initiative (WHI)]. Am J Cardiol. (2006) 97(5):730–7. 10.1016/j.amjcard.2005.09.108 16490447

[37] Zhang Y Post WS Blasco-Colmenares E Dalal D Tomaselli GF Guallar E. Electrocardiographic QT interval and mortality: a meta-analysis. Epidemiology. (2011) 22(5):660–70. 10.1097/EDE.0b013e318225768b 21709561 PMC3150395

[38] Vandenberk B Vandael E Robyns T Vandenberghe J Garweg C Foulon V QT Correction across the heart rate spectrum, in atrial fibrillation and ventricular conduction defects. Pacing Clin Electrophysiol. (2018) 41(9):1101–8. 10.1111/pace.13423 29928779

[39] Vandenberk B Vandael E Robyns T Vandenberghe J Garweg C Foulon V Which QT correction formulae to use for QT monitoring? J Am Heart Assoc. (2016) 5(6):e003264. 10.1161/JAHA.116.003264 27317349 PMC4937268

[40] Beaser AD Cifu AS. Management of patients with atrial fibrillation. JAMA. (2019) 321(11):1100–1. 10.1001/jama.2019.1264 30874742

[41] Jauch EC Saver JL Adams HP Jr Bruno A Connors JJ Demaerschalk BM Khatri P McMullan PW Jr Qureshi AI Rosenfield K Scott PA Summers DR Wang DZ Wintermark M Yonas H. Guidelines for the early management of patients with acute ischemic stroke: a guideline for healthcare professionals from the American heart association/American stroke association. Stroke 2013;44(3):870–947. 10.1161/STR.0b013e318284056a 23370205

[42] Powers WJ Derdeyn CP Biller J Coffey CS Hoh BL Jauch EC 2015 American heart association/American stroke association focused update of the 2013 guidelines for the early management of patients with acute ischemic stroke regarding endovascular treatment: a guideline for healthcare professionals from the American heart association/American stroke association. Stroke. (2015) 46(10):3020–35. 10.1161/STR.0000000000000074 26123479

[43] Ziaka M Exadaktylos A. The heart is at risk: understanding stroke-heart-brain interactions with focus on neurogenic stress cardiomyopathy—a review. J Stroke. (2023) 0(0):0–0. 10.5853/jos.2022.02173 PMC9911836 36592971

