# Supplementary material for: Corrected QTc interval combined with troponin value and mortality in acute ischemic stroke
Source: Front Cardiovasc Med. 2023 Sep 26;10:1253871. doi: 10.3389/fcvm.2023.1253871 (PMC10562700; doi:10.3389/fcvm.2023.1253871)
Supplement: Supplementary file 1 [file Table1.docx]

**Supplemental figure 1. Kaplan–Meier plots of overall survival (A and B) and survival by vascular death (C and D) and non-vascular death (E and F) according to each dichotomized ET and PQTc intervals, respectively**

ET, elevated troponin; PQTc, prolonged heart rate-corrected QT.

**p-*values determined using the log-rank test.

**
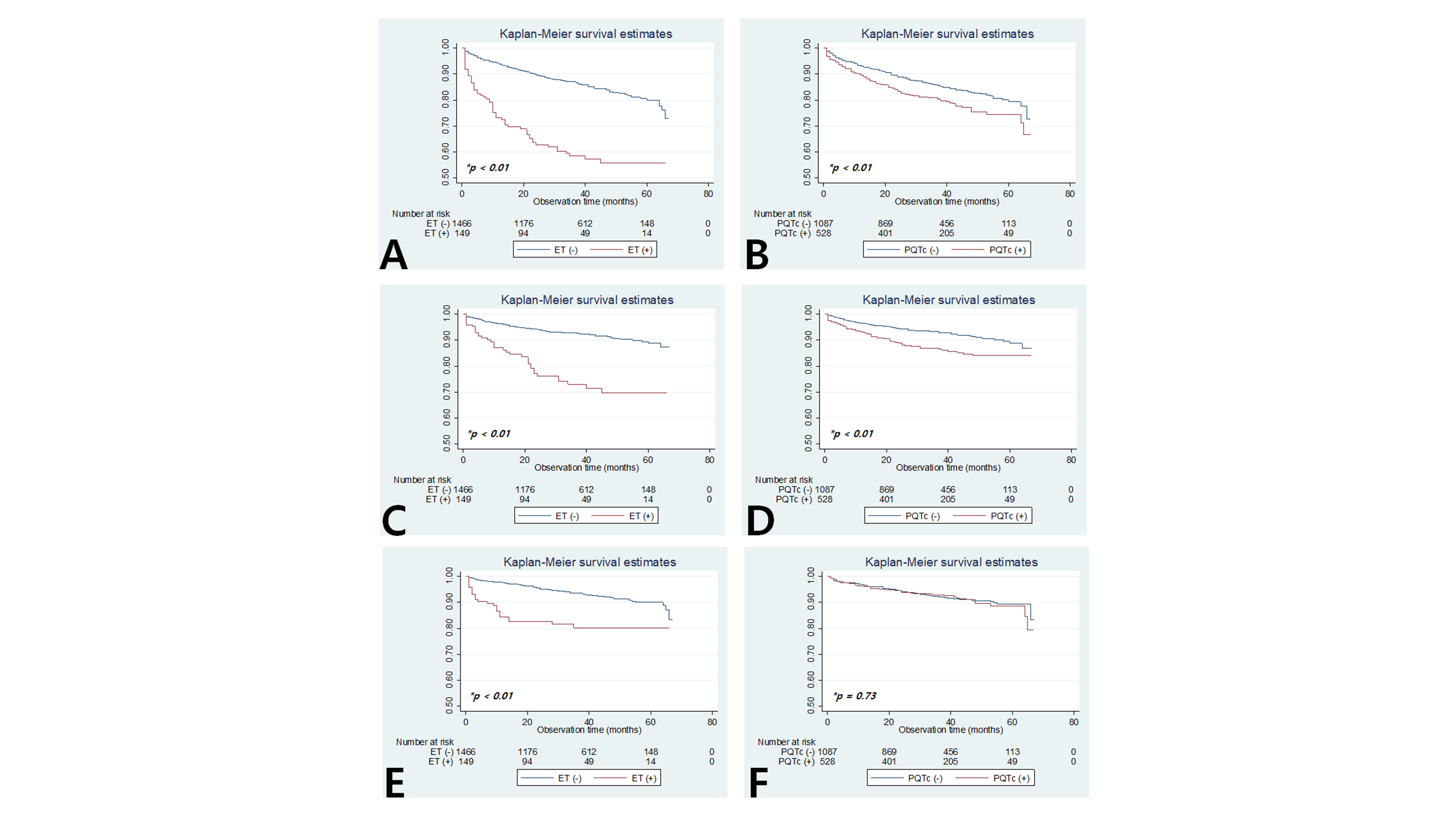
**

**Supplemental figure 2. Proportion of QTc interval according to troponin value**

**
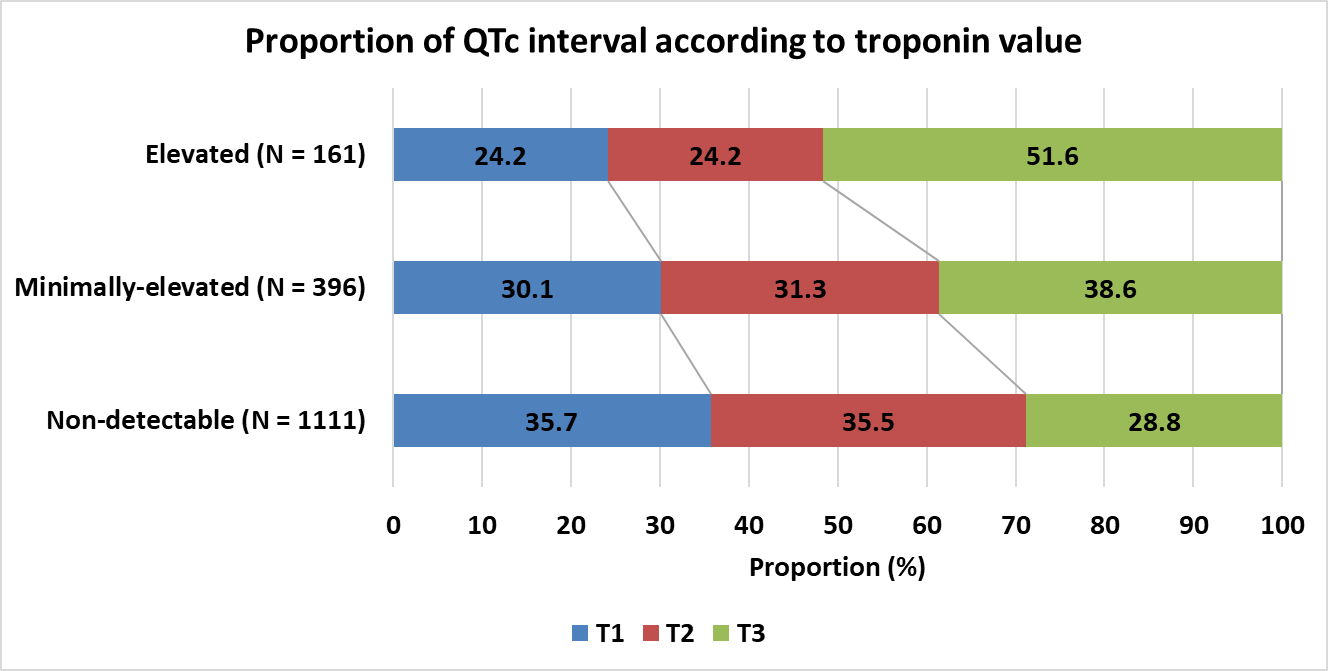
**

**Supplemental figure 3. ROC curve for an appropriate cut-off value of sex-specific QTc interval for prediction of mortality**

**
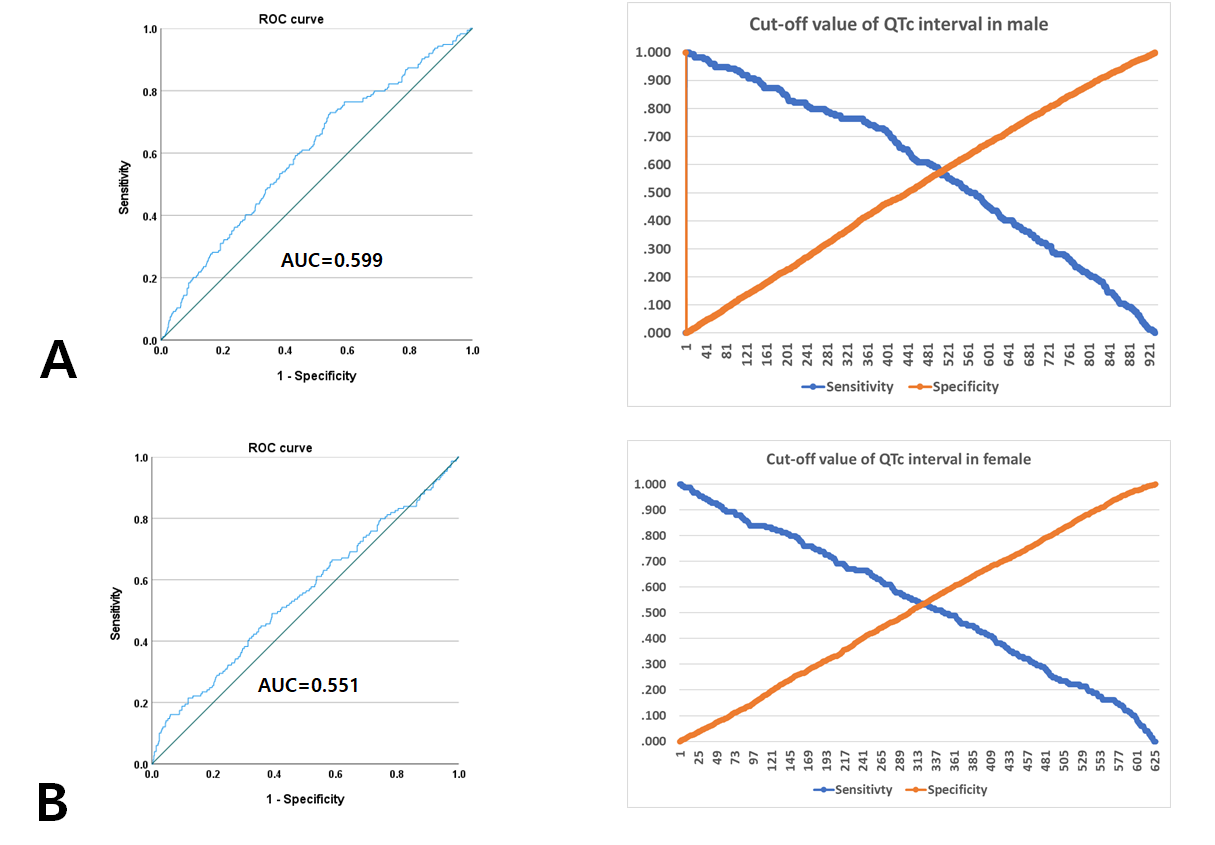
**

AUC, area under the curve; ROC, receiver operating characteristics.

**Supplemental table 1. Patient characteristics according to each dichotomized ET and PQTc interval, respectively**

|  | Stratified by troponin I level | |  |  | Stratified by QTc interval | |  |
| --- | --- | --- | --- | --- | --- | --- | --- |
| Variable | ET  (n=161) | Non-ET  (n=1507) | *p-*value^†^ |  | PQTc  (n = 556) | Non-PQTc  (n = 1112) | *p-*value^†^ |
| Age (years) | 68.6 ± 13.1 | 65.8 ± 12.3 | 0.01 |  | 67.0 ± 12.4 | 65.6 ± 12.4 | 0.03 |
| Male | 94 (58.4) | 924 (61.3) | 0.47 |  | 339 (61.0) | 679 (61.1) | 0.97 |
| **Medical history** |  |  |  |  |  |  |  |
| Hypertension | 110 (68.3) | 951 (63.1) | 0.19 |  | 377 (67.8) | 684 (61.5) | 0.01 |
| Diabetes mellitus | 36 (22.4) | 381 (25.3) | 0.42 |  | 146 (26.3) | 271 (24.4) | 0.40 |
| Hyperlipidemia | 31 (19.3) | 347 (23.0) | 0.28 |  | 123 (22.1) | 255 (22.9) | 0.71 |
| Current smoking | 44 (27.3) | 470 (31.2) | 0.31 |  | 162 (29.1) | 352 (31.7) | 0.29 |
| **Comorbidities** |  |  |  |  |  |  |  |
| AF | 69 (42.9) | 403 (26.7) | < 0.01 |  | 210 (37.8) | 262 (23.6) | < 0.01 |
| VH | 67 (41.6) | 362 (24.0) | < 0.01 |  | 164 (29.5) | 265 (23.8) | 0.01 |
| IHD | 37 (23.0) | 192 (12.7) | < 0.01 |  | 82 (14.7) | 147 (13.2) | 0.39 |
| CHF | 34 (21.1) | 139 (9.2) | < 0.01 |  | 89 (16.0) | 84 (7.6) | < 0.01 |
| CKD | 33 (20.5) | 190 (12.6) | 0.01 |  | 96 (17.3) | 127 (11.4) | < 0.01 |
| Comorbid cancer | 25 (15.5) | 63 (4.2) | < 0.01 |  | 25 (4.5) | 63 (5.7) | 0.31 |
| **Characteristics of stroke** |  |  |  |  |  |  |  |
| Previous stroke | 42 (26.1) | 391 (25.9) | 0.97 |  | 153 (27.5) | 280 (25.2) | 0.31 |
| NIHSS score | 7 [3, 14.5] | 4 [2, 9] | < 0.01 |  | 5 [2, 12] | 4 [2, 8] | < 0.01 |
| **Laboratory results** |  |  |  |  |  |  |  |
| White blood cell (10^3^/uL) | 8.6 ± 3.1 | 8.2 ± 2.8 | 0.08 |  | 8.8 ± 3.2 | 7.9 ± 2.7 | < 0.01 |
| Platelet (10^3^/uL) | 206.8 ± 87.5 | 223.9 ± 64.7 | < 0.01 |  | 224.5 ± 74.9 | 221.2 ± 63.4 | 0.36 |
| Hemoglobin (g/dL) | 13.2 ± 2.0 | 13.9 ± 1.9 | < 0.01 |  | 13.8 ± 2.1 | 13.9 ± 1.9 | 0.61 |
| Glucose (mg/dL) | 144.2 ± 52.8 | 145.5 ± 57.1 | 0.79 |  | 149.7 ± 54.0 | 143.2 ± 57.9 | 0.04 |
| Low-density lipoprotein (mg/dL) | 106.7 ± 37.5 | 108.6 ± 34.2 | 0.54 |  | 106.1 ± 34.2 | 109.5 ± 34.6 | 0.07 |
| High-density lipoprotein (mg/dL) | 43.3 ± 11.9 | 43.0 ± 11.9 | 0.76 |  | 43.3 ± 11.8 | 42.9 ± 11.9 | 0.48 |
| Albumin (g/dL) | 3.6 ± 0.5 | 3.8 ± 0.4 | < 0.01 |  | 3.8 ± 0.5 | 3.8 ± 0.4 | 0.13 |
| Homocysteine (mmol/mL) | 14.9 ± 5.8 | 14.8 ± 7.2 | 0.89 |  | 14.6 ± 5.9 | 14.9 ± 7.6 | 0.40 |
| C-reactive protein (mg/dL) | 1.1 ± 2.5 | 0.7 ± 2.1 | 0.04 |  | 0.9 ± 2.5 | 0.6 ± 1.9 | 0.03 |

Variables are presented as mean ± SD, median [interquartile range], or number (%).

AF, atrial fibrillation; CHF, congestive heart failure; CKD, chronic kidney disease; ET, elevated troponin; IHD, ischemic heart disease; NIHSS, National Institutes of Health Stroke Scale; PQTc, prolonged heart rate-corrected QT; VH, ventricular hypertrophy.

†p-values are calculated using the Pearson chi-square test, Fisher’s exact test, ANOVA test, and Kruskal Wallis test as appropriate.

***Supplemental table 2. Incidence rate per 1000 person-months and unadjusted and adjusted hazard ratios for each dichotomized ET and PQTc interval to predict clinical outcomes during the 6-year follow-up period, respectively**

| Quartiles |  |  |  |  | Unadjusted | |  | Adjusted^*^ | |  | Adjusted† | |
| --- | --- | --- | --- | --- | --- | --- | --- | --- | --- | --- | --- | --- |
|  |  | Number of events, | Incidence, % |  | HR | 95% CI |  | HR | 95% CI |  | HR | 95% CI |
| **1) Stratified by troponin value** | | |  |  |  |  |  |  |  |  |  |  |
| **All-cause mortality** |  |  |  |  |  | |  |  | |  |  | |
| Non-ET |  | 250/1507 (16.6) | 4.8 |  | Reference | |  | Reference | |  | Reference | |
| ET |  | 73/161 (45.3) | 16.8 |  | 3.31 | 2.55–4.30 |  | 2.25 | 1.67–3.04 |  | **1.83** | **1.35**–**2.47** |
| **Vascular death** |  |  |  |  |  |  |  |  |  |  |  |  |
| Non-ET |  | 145/1507 (9.6) | 2.8 |  | Reference | |  | Reference | |  | Reference | |
| ET |  | 45/161 (28.0) | 10.4 |  | 3.45 | 2.47–4.83 |  | 2.39 | 1.64–3.49 |  | **1.92** | **1.31**–**2.81** |
| **Non-vascular death** |  |  |  |  |  |  |  |  |  |  |  |  |
| Non-ET |  | 105/1507 (7.0) | 2.0 |  | Reference | |  | Reference | |  | Reference | |
| ET |  | 28/161 (17.4) | 6.4 |  | 3.11 | 2.05–4.73 |  | 1.74 | 1.02–2.95 |  | 1.58 | 0.93–2.69 |
| **2) Stratified by QTc interval** |  |  |  |  |  |  |  |  |  |  |  |  |
| **All-cause mortality** |  |  |  |  |  |  |  |  |  |  |  |  |
| Non-PQTc interval |  | 187/1112 (16.8) | 4.8 |  | Reference | |  | Reference | |  | Reference | |
| PQTc interval |  | 136/556 (24.5) | 7.7 |  | 1.55 | 1.25–1.94 |  | 1.32 | 1.03–1.69 |  | 1.08 | 0.84–1.39 |
| **Vascular death** |  |  |  |  |  |  |  |  |  |  |  |  |
| Non-PQTc interval |  | 98/1112 (8.8) | 2.5 |  | Reference | |  | Reference | |  | Reference | |
| PQTc interval |  | 92/556 (16.5) | 5.2 |  | 1.99 | 1.50–2.65 |  | 1.61 | 1.18–2.21 |  | 1.25 | 0.89–1.74 |
| **Non-vascular death** |  |  |  |  |  |  |  |  |  |  |  |  |
| Non-PQTc interval |  | 89/1112 (8.0) | 2.3 |  | Reference | |  | Reference | |  | Reference | |
| PQTc interval |  | 44/556 (7.9) | 2.5 |  | 1.07 | 0.74–1.53 |  | 1.02 | 0.68–1.54 |  | 0.96 | 0.64–1.45 |

CI, confidence interval; ET, elevated troponin; HR, hazard ratio; NIHSS, National Institutes of Health Stroke Scale; PQTc, prolonged heart rate-corrected QT.

*Model 1, adjusted for age, sex, conventional risk factors, six comorbidities, and all laboratory results in Table 1.

†Model 2, adjusted for model 1 plus the NIHSS score.

**Supplemental table 3. Medications affecting QTc interval according to the combination of ET and PQTc interval**

|  | ET (N=161) | |  | Non-ET (N=1507) | |  |
| --- | --- | --- | --- | --- | --- | --- |
| Variable | PQTc  (n=83) | Non-PQTc  (n=78) |  | PQTc  (n=473) | Non-PQTc  (n=1034) | *p-*value† |
| QTc interval prolongation |  |  |  |  |  |  |
| ACEIs or ARBs | 300 (36.1) | 31 (39.7) |  | 132 (27.9) | 308 (29.8) | 0.11 |
| Calcium channel blockers | 21 (25.3) | 26 (33.3) |  | 146 (30.9) | 265 (25.6) | 0.11 |
| Beta-blockers | 18 (21.7) | 16 (20.5) |  | 73 (15.4) | 177 (17.1) | 0.61 |
| Digoxin | 5 (6.0) | 7 (9.0) |  | 15 (3.2) | 24 (2.3) | 0.01 |
| Other QTc prolonging drugs* | 12 (14.5) | 14 (17.9) |  | 68 (14.4) | 144 (13.9) | 0.81 |
| Anti-QTc interval prolongation[^37^](#_ENREF_37) | |  |  |  |  |  |
| Statin | 18 (21.7) | 13 (16.7) |  | 92 (19.5) | 2000 (19.3) | 0.88 |

Variables are presented as numbers (%).

ACEI, angiotensin-converting enzyme inhibitor; ARB, angiotensin receptor blocker; ET, elevated troponin; PQTc, prolonged heart rate-corrected QT.

*QTc interval prolonging drugs determined by the Anatomical Therapeutic Chemical (ATC) code include cilostazol (B01AC23), domperidone (A03FA03), flecainide (C01BC04), amiodarone (C01BD01), sotalol (C07AA07), nicardipine (C08CA04), solifenacin (G04BD08), azithromycin (J01FA10), ofloxacin (J01MA01), ciprofloxacin (J01MA02), tamoxifen (L02BA01), tacrolimus (L04AD02), tizanidine (M03BX02), amantadine (N04BB01), quetiapine (N05AH04), lithium (N05AN01), risperidone (N05AX08), fluoxetine (N06AB03), citalopram (N06AB04), sertraline (N06AB06), escitalopram (N06AB10), galantamine (N06DA04), imipramine (N06AA02, N06AA02), amitriptyline (N06AA09), and diphenhydramine (R06AA02), as listed at [www.qtdrugs.org](http://www.qtdrugs.org).

†*p*-values were calculated by the Pearson chi-square test.
